# Advances in genetics and multi-omics for ischemic stroke: from pathogenesis to clinical translation

**DOI:** 10.3389/fgene.2026.1868950

**Published:** 2026-07-09

**Authors:** Siyi Zhao, Lei Liu, Junxiong Wu, Chen Long, Jun Xu, Feng Huang, Bo He, Derong Wu, Yong Liang, Chenna Yan, Lin Fu, Caimei He, Guangxiong Yuan

**Affiliations:** 1 Department of Emergency, Xiangtan Central Hospital (The Affiliated Hospital of Hunan University), Xiangtan, China; 2 School of Biomedical Sciences, Hunan University, Changsha, China; 3 Center for Reproductive and Genetic Medicine, The Central Hospital of Xiangtan (The Affiliated Hospital of Hunan University), Xiangtan, China

**Keywords:** clinical translation, genetics, ischemic stroke, mechanisms, multi-omics

## Abstract

Ischemic stroke is a multifactorial, heterogeneous cerebrovascular disease characterized by high incidence, mortality, disability, and recurrence rates. Currently, computed tomography or magnetic resonance imaging represents the gold standard for diagnosis and guidance of thrombolysis or thrombectomy. However, it has limitations such as the inability to provide rapid detection, distinguish etiologies, perform risk stratification, or enable dynamic monitoring. Therefore, rapid, convenient, and highly sensitive biomarkers hold significant clinical value. In recent years, rapid advancements in genetics and multi-omics technologies have not only driven the discovery of ischemic stroke biomarkers and the elucidation of pathophysiological mechanisms but also demonstrated broad application prospects in clinical translation—covering etiological subtyping, individual risk stratification and screening of high-risk populations, drug target identification, recurrence prediction, and prognostic assessment. These advances have laid a molecular foundation for achieving precise diagnosis and personalized treatment of ischemic stroke. This review summarizes biomarkers for ischemic stroke identified through genome-wide association study and multi-omics technologies. We also discuss the implications of these findings for the biological mechanisms of ischemic stroke and their clinical applications.

## 1 Introduction

Stroke is one of the leading causes of death and long-term disability worldwide, placing a heavy burden on public health systems and the global economy. According to the latest data from the 2021 Global Burden of Disease (GBD) study, stroke is the third leading cause of death globally, accounting for approximately 11.9 million deaths in 2021, or 10.7% of all deaths worldwide. Ischemic stroke accounted for the largest proportion of all new stroke cases (7.8 million) (Collaborators, 2024). Although significant progress has been made in acute intravascular treatments and intravenous thrombolysis, the pathophysiology of ischemic stroke is complex and involves the interaction of multiple factors, including genetics, environment, and lifestyle (Chang et al., 2023; Chojdak-Lukasiewicz et al., 2021). Significant challenges remain in identifying stroke risk, mitigating post-stroke cognitive impairment, and delivering personalized treatment. Developing new neuroprotective technologies and optimizing stroke management protocols are key to addressing these challenges. Recent breakthroughs in genetics and the multi-omics field are shedding light on how genetic and molecular factors influence stroke risk and the rehabilitation process. Recently, genetics and multi-omics have been widely used to identify biomarkers of ischemic stroke. They can dynamically and comprehensively elucidate the pathophysiological mechanisms of ischemic stroke. at multiple molecular levels, including deoxyribonucleic acid (DNA), ribonucleic acid (RNA), proteins and metabolites (Li et al., 2022).

Ischemic stroke is characterized by high heterogeneity and a significant genetic predisposition, with marked differences in the genetic basis across different subtypes (Yoshimoto et al., 2025). With the rapid advancement of high-throughput sequencing technologies, particularly large-scale, cross-ancestry genome-wide association study (GWAS) meta-analyses (such as GIGASTROKE), researchers have successfully identified nearly 100 genetic loci independently associated with stroke risk, providing important clues for understanding its pathophysiological mechanisms. Furthermore, the STROMICS genome study in the Chinese population has revealed complex genetic-phenotypic interactions among patients with ischemic stroke (Cheng et al., 2023), further underscoring the necessity of conducting genetic research in non-European populations.

However, relying solely on genomic data makes it difficult to comprehensively elucidate the dynamic evolutionary process of ischemic stroke from genetic variants to disease phenotypes. In recent years, the emergence of multi-omics technologies, including transcriptomics, proteomics, epigenomics, and metabolomics, has opened new avenues for systematically exploring the molecular mechanisms of ischemic stroke (Li et al., 2022). By integrating multidimensional omics data, researchers can not only more accurately map the cascade of reactions following cerebral ischemic injury but also identify biomarkers and therapeutic targets with potential clinical value (Li et al., 2022).

Translating findings from genetics and multi-omics research into clinical practice is the ultimate goal of achieving precision medicine for ischemic stroke. By constructing predictive models that integrate clinical risk factors with polygenic risk scores (PRS), clinicians can more accurately perform disease risk stratification and implement personalized prevention strategies (Kachuri et al., 2024). Meanwhile, the application of pharmacogenomics provides a scientific basis for optimizing antiplatelet and anticoagulant treatment regimens and reducing adverse drug reactions (Lee et al., 2026).

This review aims to systematically summarize recent research advances in the fields of genetics and multi-omics regarding ischemic stroke, focusing on the application of these technologies in elucidating disease mechanisms. Furthermore, from the perspective of the full clinical management cycle of stroke (primary prevention, acute-phase management, secondary prevention and post-stroke rehabilitation), we comprehensively evaluate the translational potential of these findings.

## 2 Genetic discoveries in ischemic stroke

Ischemic stroke is a highly heterogeneous, multifactorial disease driven by a combination of genetic susceptibility and environmental factors. Although the vast majority of cases show polygenic or sporadic characteristics, approximately 1%–5% of ischemic strokes can be attributed to monogenic mutations following Mendelian inheritance patterns (Chang et al., 2023; Chojdak-Lukasiewicz et al., 2021). Table 1 summarizes common variants associated with ischemic stroke.

**TABLE 1:** Common variants associated with ischemic stroke^*^.

​					Associations by IS subtype, p value (cross-ancestry meta-analysis)			
SNP ID	Gene (s)	Phenotype	EA (EAF)	OR (95% CI)	AIS	LAA	CE	SVO
rs2455132	*PRDM16*	SVO	C (0.73)	1.1 (1.06–1.13)	​	​	​	​
rs880315	*CASZ1*	AIS	C (0.45)	1.05 (1.03–1.06)	​	​	​	​
rs3790607	*WNT2B*	AIS	C (0.26)	1.05 (1.04–1.07)	​	​	​	​
rs2251636	*PMF1*	SVO	G (0.62)	1.09 (1.06–1.12)	​	​	​	​
rs680084	*PRRX1*	CE	G (0.47)	1.1 (1.07–1.13)	​	​	​	​
rs11694327	*KCNK3*	AIS	C (0.39)	1.04 (1.03–1.05)	​	​	​	​
rs2351524	*NBEAL1*	AIS	C (0.88)	1.07 (1.05–1.09)	​	​	​	​
rs73852583	*3p12*	AIS	A (0.10)	1.41 (1.25–1.6)	​	​	​	​
rs16998073	*FGF5*	AIS	T (0.30)	1.04 (1.03–1.06)	​	​	​	​
rs6847935	*PITX2*	CE	T (0.26)	1.32 (1.28–1.37)	​	​	​	​
rs6536024	*FGG*	CE	C (0.54)	1.05 (1.04–1.07)	​	​	​	​
rs4444878	*F11*	CE	A (0.39)	1.1 (1.07–1.13)	​	​	​	​
rs17148926	*LOC100505841*	AIS	A (0.83)	1.07 (1.05–1.08)	​	​	​	​
rs79318212	*FOXF2*	AIS	G (0.11)	1.09 (1.07–1.11)	​	​	​	​
rs36229526	*TAP1*	AIS	T (0.08)	1.05 (1.02–1.08)	​	​	​	​
rs2501966	*CENPQ*	AIS	A (0.54)	1.04 (1.03–1.05)	​	​	​	​
rs56393506	*LPA*	LAA	T (0.16)	1.16 (1.1–1.22)	​	​	​	​
rs2107595	*HDAC9*	LAA	A (0.23)	1.16 (1.12–1.21)	​	​	​	​
rs42035	*CDK6*	AIS	A (0.76)	1.06 (1.04–1.07)	​	​	​	​
rs12539561	*PIK3CG*	AIS	C (0.17)	1.04 (1.03–1.06)	​	​	​	​
rs114798023	*THAP5*	INC_AIS	G (0.02)	1.07 (1.02–1.12)	​	​	​	​
rs1549758	*NOS3*	AIS	T (0.27)	1.04 (1.03–1.06)	​	​	​	​
rs2738158	*DEFB1*	CE	G (0.21)	1.14 (1.09–1.19)	​	​	​	​
rs1487504	*BNC2*	AIS	A (0.11)	1.05 (1.03–1.07)	​	​	​	​
rs7859362	*CDKN2B-AS1*	AIS	C (0.54)	1.04 (1.03–1.05)	​	​	​	​
rs649129	*ABO*	AIS	T (0.22)	1.06 (1.04–1.08)	​	​	​	​
rs55983834	*SH3PXD2A*	AIS	C (0.55)	1.05 (1.04–1.07)	​	​	​	​
rs10886430	*GRK5*	AIS	G (0.12)	1.08 (1.05–1.1)	​	​	​	​
rs60401382	*HTRA1*	SVO	C (0.64)	1.08 (1.05–1.12)	​	​	​	​
rs1973765	*LSP1*	AIS	C (0.48)	1.04 (1.02–1.05)	​	​	​	​
rs415895	*SWAP70*	AIS	G (0.58)	1.04 (1.02–1.05)	​	​	​	​
rs72985562	*MMP12*	LAA	G (0.08)	1.23 (1.14–1.33)	​	​	​	​
rs7304841	*PDE3A*	AIS	A (0.60)	1.05 (1.03–1.06)	​	​	​	​
rs12426667	*HOXC4*	AIS	A (0.65)	1.04 (1.03–1.05)	​	​	​	​
rs10774625	*SH2B3*	AIS	A (0.47)	1.07 (1.05–1.08)	​	​	​	​
rs7974266	*PTPN11*	AIS	T (0.60)	1.04 (1.03–1.06)	​	​	​	​
rs35429	*12q24*	AIS	A (0.64)	1.04 (1.02–1.05)	​	​	​	​
rs842365	*LRCH1*	AIS	A (0.73)	1.04 (1.03–1.06)	​	​	​	​
rs9515201	*COL4A2*	SVO	A (0.25)	1.11 (1.07–1.15)	​	​	​	​
rs1573644	*FURIN*	AIS	C (0.31)	1.04 (1.03–1.06)	​	​	​	​
rs2397816	*LINC00924*	AIS	A (0.41)	1.04 (1.03–1.05)	​	​	​	​
rs2359171	*ZFHX3*	CE	A (0.19)	1.17 (1.13–1.21)	​	​	​	​
rs12445022	*ZCCHC14*	SVO	A (0.26)	1.11 (1.07–1.15)	​	​	​	​
rs2108911	*MTMR4*	AIS	C (0.20)	1.05 (1.04–1.07)	​	​	​	​
rs28860769	*SLC44A2*	AIS	G (0.79)	1.05 (1.03–1.07)	​	​	​	​
rs8106503	*LDLR*	AIS	T (0.89)	1.06 (1.04–1.09)	​	​	​	​
rs11907011	*PROCR*	AIS	C (0.90)	1.06 (1.04–1.09)	​	​	​	​

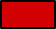
, P < 5 × 10^−8^; 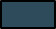, P < 5 × 10^−5^; 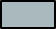, P < 5 × 10^−2^; 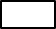, P > 5 × 10^−2^. Abbreviation: A, adenine; AIS, any ischemic stroke; C, cytosine; CE, cardioembolic stroke; CI, confidence interval; EA, effect allele; EAF, effect allele frequency; G, guanine; INC_AIS, incident any stroke; LAA, large artery atherosclerosis; OR, odds ratio; SNP, single nucleotide polymorphism; SVO, small-vessel occlusion; T, thymine.

* Top significant signals from previous cross-ancestry genome-wide association study meta-analyses in GIGASTROKE (all rows apart from any stroke) (Mishra et al., 2022). Loci shown meet genome-wide significance (*P* < 5 × 10^−8^).

### 2.1 Monogenic ischemic stroke

Among monogenic cerebral small-vessel diseases, Cerebral autosomal dominant arteriopathy with subcortical infarcts and leukoencephalopathy (CADASIL) is the most common type, with an estimated global prevalence of approximately 2–5 cases per 100,000 people (Yuan et al., 2024). CADASIL is primarily caused by missense mutations in the *NOTCH3*, located on chromosome 19p13 (Cheng et al., 2023; Di Donato et al., 2017). This leads to progressive degeneration of vascular smooth muscle cells and the formation of characteristic granular osmophilic material (GOM) deposits in the basement membrane. Other important monogenic disorders include cerebral small vessel diseases caused by mutations in the *COL4A1* and *COL4A2*, which encode type IV collagen, a key component of the vascular basement membrane (Bordes et al., 2022). Mutations in these genes lead to vascular wall fragility and increase the risk of lacunar strokes and cerebral microbleeds. Moreover, sequencing has further revealed the pathogenic role of heterozygous mutations in autosomal dominant cerebral small vessel diseases. Unlike the homozygous mutations that cause classic CARASIL (autosomal recessive), heterozygous *HTRA1* mutations typically present in late adulthood (>45 years) with ischemic stroke and progressive cognitive decline (Malik et al., 2021; Verdura et al., 2015). With advances in genetic technologies, an increasing number of rare monogenic causes involving different cell types have been reported, further highlighting the complexity of ischemic stroke. For example, mutations in the *CSF1R* not only cause adult-onset leukoencephalopathy with axonal spheroids and pigmented glia but also profoundly affect the survival and homeostasis of microglia, suggesting the indispensable role of resident immune cells in the central nervous system in maintaining cerebral vascular integrity (Wu et al., 2023). In addition, rare variants in genes such as *ARHGEF15* (Ding et al., 2023) and *PLOD3* (Zhou et al., 2022) have also been confirmed to be associated with familial cerebral small-vessel disease.

### 2.2 Polygenic diseases

In contrast to rare monogenic mutations, the vast majority of ischemic stroke cases exhibit highly complex polygenic inheritance patterns, with polygenic diseases accounting for approximately 38% of all ischemic stroke cases (Fox et al., 2003).

In recent years, GWAS has become a core tool for exploring the polygenic genetic architecture of ischemic stroke. With deepening of international collaboration, the sample size for ischemic stroke GWAS has grown significantly. In 2018, MEGASTROKE successfully identified 32 stroke-specific risk loci through a meta-analysis of approximately 520,000 participants (Malik et al., 2018). In 2022, GIGASTROKE published the largest cross-ancestry GWAS meta-analysis to date, including over 2.5 million participants and covering five major ancestral groups. This study not only confirmed previous findings but also identified 61 new independent stroke risk loci, bringing the total number of known stroke-associated risk loci to 89 (Mishra et al., 2022).

Notably, cross-ancestry analysis revealed that approximately 87% of the major stroke risk loci share genetic effects across different populations, suggesting that the core pathophysiological mechanisms of stroke are highly conserved across races (Mishra et al., 2022). However, some loci also show significant ancestral specificity. For instance, the p.R4810K variant in the *RNF213* gene is significantly more prevalent in East Asian populations than in European populations (Zhou et al., 2023; Kim, 2016). A large-scale study of 46,958 Japanese individuals found that carriers of this variant have a 3.6-fold higher risk of large-artery atherosclerotic stroke than non-carriers (Mineharu and Miyamoto, 2021). COMPASS found that the *HNF1A* variant (rs55931441) exhibits significant specificity to African populations, whereas this variant is virtually absent in European populations (Keene et al., 2020). Furthermore, in Indian populations (Kumar et al., 2021) and in cross-ancestry studies targeting ischemic stroke risk factors such as atrial fibrillation (AF) (Miyazawa et al., 2023), multiple risk genes specific to non-European populations have been successively identified (e.g., *SLC17A2*, *FAM73A*, *OR52L1*, *SYNE1*, and *FGF13*) (Miyazawa et al., 2023; Kumar et al., 2021). With improved research methods and increasing availability of multi-ancestry cohorts, more valuable stroke-related risk genes are expected to be identified in the future, thus advancing population-transcending precision prevention.

### 2.3 The blurring boundary between monogenic and multifactorial stroke

In traditional genetic understanding, monogenic and multifactorial stroke have been regarded as two distinct categories. However, with the progress of large-scale genomic studies, this distinction is becoming increasingly blurred. Monogenic stroke is considered rare, but recent studies report that pathogenic variants are more common in the general population (Rutten et al., 2019). For example, a typical *NOTCH3* variant is found in 1 in 452 people (Cho et al., 2021), an *HTRA1* variant in 1 in 275 people (Malik et al., 2021), and a *COL4A1*/*2* variant in 1 in 1,353 people (Cho et al., 2022). The clinical relevance of these seemingly asymptomatic mutations remains unclear. Nevertheless, one study indicates that these mutations are associated with an increased risk of stroke and vascular dementia, as well as an increased prevalence of cerebral small vessel disease magnetic resonance imaging markers. The burden of cardiovascular risk correlates with the penetrance of these variants. *NOTCH3* and *HTRA1* variants are associated with increased white matter volume and reduced white matter integrity. *NOTCH3* variants are also associated with increased brain volume, although the mechanism remains unclear (Cho et al., 2022). In recent years, the clinical phenotype of CADASIL has been shown to vary widely, some individuals experience stroke at a young age, while others do not develop stroke until their 80s (Hack et al., 2022). Several factors have been proposed to modulate the phenotype, including the location of the variant (variants in epidermal growth factor-like repeat sequences [EGFRs] 1–6 are associated with more severe disease) (Cho et al., 2022; Cheng et al., 2023; Rutten et al., 2019), the presence of cardiovascular risk factors (Cho et al., 2022; Adib-Samii et al., 2010) and modifier genes (Opherk et al., 2006). Similar effects have been observed in *HTRA1*, with the p.Arg227Trp variant associated with a lower risk of stroke (Cho et al., 2022). Therefore, monogenic small-vessel occlusion mutations appear far more common than previously recognized and may contribute to the burden of seemingly sporadic small-vessel occlusion.

## 3 The biological mechanisms of ischemic stroke

While GWAS have implicated hundreds of loci in ischemic stroke, they often do not shed light on the biological mechanism or establish causality. To close this knowledge gap, genetics and multi-omics are increasingly being applied to translate associations into functional mechanisms.

### 3.1 Genetics of ischemic stroke

**FIGURE 1:**
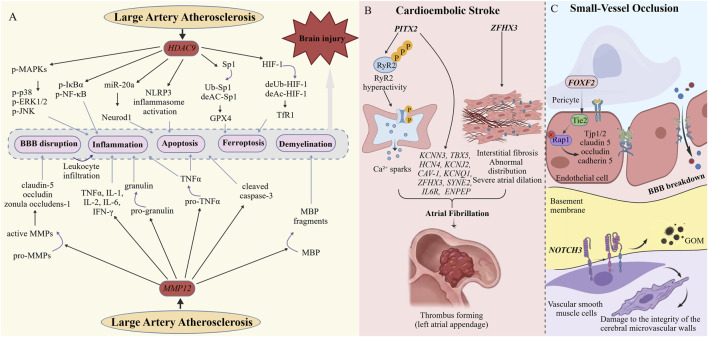
Mechanism diagram of Three Main Subtypes of Ischemic Stroke. **(A)** Schematic model demonstrating the roles of *HDAC9* and *MMP12* in large artery atherosclerosis. **(B)** Schematic model demonstrating the roles of *PITX2* and *ZFHX3* in cardioembolic stroke. **(C)** Schematic model showing the role of *FOXF2* and *NOTCH3* in small-vessel occlusion. Abbreviation: Ub, ubiquitination; deUb, deubiquitination; deAc, deacetylation; Tjp1/2, tight junction protein 1/2; RyR2, ryanodine receptor 2. This figure was created with BioGDP.com (Ventura Jiang et al., 2025).

This section reviews the latest findings on genetic and molecular mechanisms for the three major subtypes, large artery atherosclerosis, cardioembolic stroke, and small-vessel occlusion, with a focus on key genes and loci of current research interest. Figure 1 summarizes mechanism diagram of three main subtypes of ischemic stroke.

#### 3.1.1 Large artery atherosclerosis

*HDAC9* is one of the most critical risk genes for large artery atherosclerosis. As a histone deacetylase, it regulates the transcriptional process by catalyzing the removal of acetyl groups from lysine residues in histones and non-histone proteins. Overexpressed in neurons during stroke, it suppresses autophagy and activates the inhibitor kappa B alpha/nuclear factor kappa-B (IκBα/NF-κB) and mitogen-activated protein kinase (MAPK) signaling pathways, reducing miR-20a expression to upregulate its target gene neuronal differentiation 1 (NeuroD1) (Huang et al., 2025), deacetylating hypoxia-inducible factor_1 (HIF-1) and specificity protein 1 (Sp1) to promote transferrin receptor 1 (TfR1) transcription and reduce glutathione peroxidase 4 (GPX4) (Sanguigno et al., 2023), and binding to and mediating the deacetylation of the NOD-like receptor family pyrin domain-containing 3 (NLRP3) to activate the inflammasome (Asare et al., 2025), thereby exacerbating neuronal damage following ischemia-reperfusion. Broad-spectrum HDAC inhibitors have contraindications that limit their application in cardiovascular diseases (Asare et al., 2025). For example, treatment with trichostatin A exacerbates atherosclerotic plaque formation, despite its inhibitory effect on macrophage function. Developing drugs capable of specifically inhibiting *HDAC9* overexpression or activity could help overcome this limitation and offer advantages over interleukin-1β (IL-1β) blockade (Asare et al., 2025). First, *HDAC9* specifically binds to NLRP3 but does not bind to other inflammasome sensors involved in IL-1β production, such as those recognizing pathogens. Therefore, targeted inhibition of *HDAC9* is not expected to substantially impair the body’s antimicrobial immune capacity. Second, inhibiting *HDAC9* reduces IL-18 and pyroptotic cell death, thereby providing additional anti-atherogenic protection. Third, while *HDAC9* plays a pro-atherogenic role in both early and late atherosclerosis, IL-1β exhibits certain protective functions in late-stage plaques; consequently, the therapeutic window for *HDAC9* inhibition is broader. Current studies have explored strategies using lipoprotein nanoparticles to specifically deliver class IIa HDAC inhibitors to bone marrow-derived cells, achieving plaque stabilization in animal models (Asare et al., 2025). This approach opens new avenues for precision therapy, addressing the clinical limitations of broad-spectrum HDAC inhibitors caused by off-target effects (such as exacerbating plaque formation).

GWAS studies have shown that the rs660599 variant in the *MMP12* gene is significantly associated with susceptibility to large artery atherosclerosis (Mishra et al., 2022). During the development of atherosclerosis, elevated levels of MMP12 in the brain promote apoptosis, inflammation, disruption of the blood-brain barrier, demyelination, and white matter damage (Veeravalli, 2024). Furthermore, activated MMP12 can activate various MMPs, which collectively contribute to the pathogenesis of large artery atherosclerosis (Veeravalli, 2024). Despite extensive research, translating MMP-targeting strategies into effective clinical treatments remains challenging (Tanase et al., 2025). First-generation broad-spectrum MMP inhibitors failed to meet clinical expectations due to off-target effects, such as musculoskeletal complications and impaired tissue regeneration. Consequently, efforts have shifted toward developing more selective inhibitors that target specific MMPs while avoiding interference with other MMPs critical for tissue stability, making this a key research direction for personalized diagnosis and treatment.

#### 3.1.2 Cardioembolic stroke

Genetic susceptibility to cardioembolic stroke is highly dependent on AF, and its core risk genes, *PITX2* and *ZFHX3*, represent two complementary yet mechanistically distinct genetic pathways underlying AF pathogenesis: electrophysiological remodeling and structural remodeling. Electrophysiological remodeling is primarily driven by *PITX2*. Haploinsufficiency induced by the *PITX2* risk allele has been shown to cause hyperphosphorylation of ryanodine receptor 2 and an increase in calcium sparks within atrial myocytes. Consequently, ectopic pacemakers that trigger AF are formed (van der Maarel et al., 2025; Jimenez-Sabado and Hove-Madsen, 2025; Kim et al., 2023). In contrast, structural remodeling is predominantly mediated by *ZFHX3*. Specific knockout of ZFHX3 in animal models leads to severe atrial dilatation, interstitial fibrosis, and the abnormal distribution of gap junction proteins. The synchronized conduction of electrical signals is not only impeded by this structural disruption, but atrial contractile function is also severely impaired, thereby substantially increasing the risk of left atrial appendage thrombus formation (Park et al., 2025; Jameson et al., 2023). The effects of PITX2 and ZFHX3-associated variants on cardioembolic stroke are primarily indirectly mediated through AF and its associated atrial pathologies; as for whether these variants have direct cerebrovascular effects independent of AF, there is currently a lack of sufficient evidence from human cerebrovascular tissue-specific expression quantitative trait locus (eQTL), functional experiments, or mendelian randomization (MR) studies to support this. Future research needs to further distinguish between AF-dependent pathways and potential AF-independent pathways. Beyond the AF pathway, a strong genetic association has been identified between cardioembolic stroke and venous thromboembolism. Genic loci located near *FGA* (encoding fibrinogen alpha), *FGG* (encoding fibrinogen gamma), *F11* (encoding coagulation factor XI), and *PROCR* (encoding protein C receptor) are established risk loci for venous thromboembolism and susceptibility loci for cardioembolic stroke; they are known to be associated with coagulation (Daghlas et al., 2025). Three distinct pathways for future precision medicine have been illuminated by genetic discoveries concerning cardioembolic stroke. First, genotype-based personalized antiarrhythmic therapy: for instance, in patients with PITX2 mutations, the development of specific inhibitors targeting ryanodine receptor 2 (e.g., ent-Verticilide) may prove more efficacious than conventional antiarrhythmic drugs (Kim et al., 2023). Second, the development of novel anticoagulants guided by genetic targets: F11 (factor XI), serving as a potent genetic risk factor for cardioembolic stroke, has emerged as a highly promising novel target for anticoagulation. Currently, phase II clinical trials are being conducted to evaluate F11 and F11a inhibitors (such as abelacimab, BAY 2433334, and BMS-986177) for the primary or secondary prevention of stroke (NCT04755283, NCT04304508, NCT03766581) (Mishra et al., 2022). Third, neurovascular protective strategies targeting the protein C receptor pathway: a recombinant variant of human activated protein C that binds to the protein C receptor (3K3A-APC, NCT02222714) has been demonstrated in phase I and II trials to be effective for the treatment of acute ischemic stroke following thrombolysis, mechanical thrombectomy, or both, and preparations are currently underway for an upcoming phase III trial (Mishra et al., 2022).

#### 3.1.3 Small-vessel occlusion

Recently identified genetic factors, such as *FOXF2* (Traylor et al., 2021; Markus and Joutel, 2025), have been shown to disrupt the blood-brain barrier (BBB) by influencing the pericytic-endothelial axis, making them key targets in the pathogenesis of small-vessel occlusion. *FOXF2*, a major risk gene for small vessel occlusion, maintains endothelial cell (EC) function through Tie2 signaling. Pharmacological targeting of EC-specific signaling pathways, such as the Tie2 agonist AKB-9778, can improve stroke outcomes, reduce infarct volume, and prevent blood-brain barrier leakage (Todorov-Volgyi et al., 2026). AKB-9778 has been tested in clinical trials for eye diseases and has demonstrated good tolerability (Campochiaro et al., 2016). However, whether long-term pharmacological activation of the Tie2 signaling pathway can have a positive effect on neurovascular function warrants further investigation.

Mutations in the *NOTCH3* gene cause CADASIL. These mutations are highly concentrated in the EGFR domain of the *NOTCH3* receptor extracellular region, leading to changes in the number of cysteine residues (Rutten et al., 2014). This triggers abnormal folding and polymerization of the *NOTCH3* receptor extracellular domain (Rutten et al., 2014; Monet-Lepretre et al., 2013), resulting in the degeneration of vascular smooth muscle cells and the formation of characteristic granular osmophilic material deposits in basement membrane. This triggers a cascade of events that disrupts the cerebral microvascular wall integrity, thereby reducing cerebral vascular reactivity (van den Brink et al., 2023) and cerebral perfusion (van den Boom et al., 2003). Combined with findings regarding other risk genes such as *COL4A1*/*2* (Bordes et al., 2022; Joutel et al., 2016), *LOX*, *SH3PXD2A*, *GPR126* and *HTRA1* (Traylor et al., 2021), the genetic pathophysiology of small-vessel occlusion points to a common pathway involving impaired microvascular extracellular matrix (ECM) remodeling and loss of the BBB.

### 3.2 Multi-omics of ischemic stroke

Although genetics provides a static “blueprint” for susceptibility to ischemic stroke, disease onset and progression are dynamic processes strongly influenced by the environment. The introduction of multi-omics technologies has enabled researchers to comprehensively analyze the pathophysiological networks of ischemic stroke from multiple dimensions, including epigenetics, transcription, protein expression, and metabolites, and to identify dynamic biomarkers with high clinical translational value.

#### 3.2.1 Epigenomics

Epigenetic modifications refer to processes that alter gene expression without changing the DNA sequence (Zhang et al., 2018). DNA methylation is a fundamental epigenetic mechanism that governs cellular reprogramming and the maintenance of physiological cell functions. DNA methylation is a dynamic process, primarily regulated by the antagonistic interaction between DNA methyltransferases (DNMTs) and ten-eleven translocation dioxygenases (TET) (Guo Y. et al., 2025). Following ischemic brain injury, profound alterations in the gene expression profile occur, which are critical for initiating cellular repair within the ischemic penumbra. Specifically, ischemic stroke triggers a pathological elevation in global DNA methylation, which suppresses the transcription of essential neuroprotective genes. This transcriptional silencing exacerbates ischemic brain damage and is strongly correlated with aberrant hyperactivation of DNMTs (Guo Y. et al., 2025). Within this cascade, TET proteins play an indispensable role in regulating the dynamic equilibrium between 5-hydroxymethylcytosine and 5-methylcytosine, which is essential for preserving genomic integrity (Guo Y. et al., 2025). In addition to DNA methylation, histone post-translational modifications—particularly histone acetylation—represent another pivotal epigenetic regulatory layer within the neuroinflammatory microenvironment post-stroke. The homeostatic level of histone acetylation is finely tuned by the opposing enzymatic activities of histone acetyltransferases (HATs) and histone deacetylases (HDACs). HATs catalyze the transfer of acetyl groups to lysine residues on histone tails, neutralizing their positive charges and promoting chromatin relaxation, which facilitates transcriptional activation. In contrast, HDACs are typically upregulated following ischemic stroke, leading to the transcriptional repression of pro-survival factors and the concomitant activation of pro-inflammatory cascades (Guo Y. et al., 2025). Pharmacological interventions targeting these epigenetic modifiers hold significant therapeutic promise for correcting pathological epigenetic landscapes and restoring cellular homeostasis. Notably, small-molecule agents such as Tubastatin A (selective HDAC6 inhibitor), Lys-CoA (HAT inhibitor), and vitamin C (TET activator) have demonstrated substantial efficacy in ameliorating stroke-induced neuropathology (Guo Y. et al., 2025).

Furthermore, environmental risk factors, most notably aging and hypertension, can induce profound epigenetic reprogramming that accelerates the progression of cerebrovascular disease. Against the backdrop of aging, the post-stroke BBB methylome and transcriptome exhibit three distinct signatures of aberrant epigenetic regulation: angiogenesis, structural proteins, and endothelial-to-mesenchymal transition (Phillips et al., 2023). Similarly, chronic hypertension alters the epigenetic landscape of the vasculature. DNA hypomethylation of blood pressure-associated genes, including *PRDM6*, *IGFBP3*, *PDE3A*, and *HDAC9*, is strongly correlated with an elevated risk of ischemic stroke and poor functional recovery (Zhang et al., 2022). These persistent epigenetic alterations disrupt the homeostasis of vascular smooth muscle and endothelial cells, ultimately rendering the cerebral vasculature highly vulnerable to ischemic insults. In the field of epigenetics, the use of whole blood samples to detect DNA methylation has significant limitations. Although the methylation profiles of blood and brain tissue show a strong correlation in certain biological processes, blood samples are unable to detect specific mechanisms within brain tissue. Furthermore, due to clinical constraints, it is difficult to obtain brain tissue samples from survivors of acute stroke.

#### 3.2.2 Transcriptomic

Transcriptomics is a discipline that systematically studies all RNA transcripts in an organism and their expression levels, using high-throughput technologies to comprehensively analyze the gene expression profiles of specific cells, tissues, or biological systems under specific spatiotemporal conditions. Based on their functional properties, transcripts can be divided into two major categories: coding RNAs, which can be translated into proteins, and non-coding RNAs, which influence gene expression through post-transcriptional regulation. Microarrays or RNA sequencing are used to analyze all known coding and non-coding transcripts. Table 2 summarizes the major potential ischemic stroke biomarkers found in transcriptomics studies.

**TABLE 2:** Potential ischemic stroke biomarkers identified from transcriptomic studies.

RNA type	Biomarkers	Sample	Expression	Interaction	Process	Refs
lncRNA	*MEG3*	MCAO mouse model/OGD/R-induced N2a cells	Up	miR-424-5p/Sema3A	Apoptosis and inflammation	Xiang et al. (2020)
		MCAO rat model/OGD/R-induced SK-N-SH and SH-SY5Y cells	Up	miR-485/AIM2	Apoptosis and inflammation	Liang et al. (2020)
	*SNHG14*	MCAO rat model/OGD-induced primary cortical neuron	Up	miR-181c-5p/BMF	Apoptosis and inflammation	Bu et al. (2021)
		OGD-induced BV2 cells	Up	miR-199b/AQP4	Apoptosis and inflammation	Zhang et al. (2021a)
	*XIST*	MCAO mouse model/OGD/R-induced bEnd3 cells	Down (early)/Up (late)	miR-92a/Itgα5 or KLF4	Angiogenesis	Wang et al. (2021)
	*MIAT*	50 patients with ischemic stroke, MCAO mouse model	Up	miR-874-3p/IL1B	Apoptosis and inflammation	Zhang et al. (2021b)
	*MALAT1*	CI/RI rat model/OGD/R -induced PC12 cells	Up	miR-375/PDE4D	Apoptosis and inflammation	Zhang et al. (2020)
miRNA	miR-21	MCAO mouse model/OGD/R-induced cortical neurons	Down	p53/Bcl-2/Bax	Apoptosis	Yan et al. (2021)
	miR-21-5p	60 patients with IS, OGD-induced HMEC-1 and 293T cells	Down	IL-6R	Apoptosis and inflammation	Zhan et al. (2023)
	miR-29b	MCAO mouse model, PC12 cells	Up	PI3K/Akt/Bax	Apoptosis, oxidative stress	Ma et al. (2022)
circRNA	miR-140-5p	MCAO mouse model/OGD-induced primary cortical neuron	Down	TLR4/NF-κB	Apoptosis	Song et al. (2021)
	circCTNNB1	MCAO mouse model/OGD/R-induced primary mouse astrocytes	Down	miR-96-5p/SRB1	Apoptosis, oxidative stress and inflammation	Chen et al. (2022)
	circPTP4A2	tMCAO mouse model/OGD/R-induced primary mouse microglia and BV2 microglial cells	Up	STAT3	Neuroinflammation	Wang et al. (2025b)
	circPDS5B	pMCAO, OGD-induced HBMECs cells	Up	miRNA-223-3p/NOTCH2	Angiogenesis	Kui et al. (2023)
mRNA	Angptl4	tMCAO mouse mode	Up	VEGFR2-Src	Angiogenesis	Bouleti et al. (2013)
	Angpt2	pMCAO and tMCAO mouse model	Up	Tie2	Angiogenesis	Gurnik et al. (2016)
	NAMPT	Hind-limb ischaemic model	Up	NAD–SIRT1	Angiogenesis	Wang et al. (2015)

Abbreviation: CI/RI, cerebral ischemia/reperfusion injury; OGD/R, oxygen-glucose deprivation/reoxygenation; MCAO, middle cerebral artery occlusion; pMCAO, permanent middle cerebral artery occlusion; tMCAO, transient middle cerebral artery occlusion.

The biological mechanisms underlying cerebral ischemia and stroke recovery are closely linked to RNA. Angiopoietin-like protein 4 (Angptl4) and angiopoietin-2 (Ang-2) are overexpressed following ischemic stroke. Both influence brain injury by regulating angiogenesis, vascular stability, and vascular permeability. Among these, numerous clinical studies have demonstrated that Angptl4 promotes angiogenesis and neurogenesis for post-stroke treatment by reducing neuronal death and inflammatory responses (Luo et al., 2023). Furthermore, whole-transcriptome analysis revealed that inflammatory and innate immune pathways, including IL-6, IL-1, STAT3, S100A12, MMP9, NF-κB, Toll-like receptors, PI3K/AKT, BCL2A1, FAM200B, IGF-1, and TXN-as-IS, are upregulated following ischemic stroke (Amini et al., 2023; Liu et al., 2025). Concurrently, these messenger RNAs (mRNAs) serve as key target genes for non-coding RNAs. Long non-coding RNAs (lncRNAs) and circular RNAs (circRNAs) act as molecular “sponges,” competitively binding to miRNAs to prevent their degradation or translational inhibition of target mRNAs. For example, miR-21-5p can specifically target and inhibit the expression of IL-6R, thereby reducing apoptosis (Zhan et al., 2023); lncRNA MIAT impairs neurological function in ischemic stroke via up-regulating miR-874-3p-targeted IL1B (Zhang S. et al., 2021); circRNA *PTP4A2* has been shown to stimulate neuroinflammation by driving STAT3-dependent microglial polarization in ischemic brain injury. This may represent a novel therapeutic target for ischemic stroke (Wang et al., 2024). Among these ncRNAs, lncRNA *MALAT1*, *MEG3*, *SNHG14*, and *MIAT* are key molecules regulating post-stroke inflammatory cascades and cell death (Zhang et al., 2020; Xiang et al., 2020; Zhang G. et al., 2021; Zhang S. et al., 2021; Bu et al., 2021). Mechanistic studies have shown that knocking down these lncRNAs significantly reduces the release of pro-inflammatory cytokines, decreases apoptosis, and improves scores of neurological deficits. This complex competitive endogenous RNA (ceRNA) network constitutes the core of transcriptional regulation in ischemic brain injury and provides a theoretical foundation for the development of multi-target therapeutic strategies. However, the transcriptome exhibits significant temporal dynamics. Biomarkers that are highly diagnostic during the hyperacute phase may see their signals disappear entirely or their expression profiles reverse within 24 h. Most human studies rely on cross-sectional sampling and thus fail to capture these temporal trajectories.

#### 3.2.3 Proteomics

Proteomics enables the systematic qualitative and quantitative analysis of the entire proteome under specific conditions (Li et al., 2022; Aslam et al., 2017). Compared to genomics or transcriptomics, proteomics is more closely linked to phenotypes and can directly reflect the dynamic changes in protein expression and functional performance under conditions of ischemic injury. It has become an attractive target for the study of biomarkers for ischemic stroke. The main possible biomarkers of ischemic stroke found in proteomics research are summarized in Table 3.

**TABLE 3:** Potential ischemic stroke biomarkers identified from proteomics studies.

Protein	Sample	Platform	Expression	Validation status	Cohort size	AUC^†^	Function	Findings	Limitations	Refs
APOB, SERPINA1	Serum	SWATH-MS	Up	No validation	80	​	Clot formation, inflammation	The first label-free proteomic study that identified blood biomarkers for the diagnosis of stroke within 24 h of symptom onset.	Lack of validation queue, insufficient sample size	Misra et al. (2022)
F2, FGA, PLG, HRG	Serum	DIA, MRM	Up	Single-center internal validation	34/61*	>0.9	Coagulation cascade	This helps diagnose strokes more accurately and quickly	Insufficient sample size, incomplete adjustment for confounding factors	Lee et al. (2020a)
F2, F9, PLG, FN1, VTN, HRG, PROS1, THBS-1, C1s, GPX3	Serum	SWATH-MS	Up	Single-center internal validation	40/120*	>0.9	Coagulation cascade, complement pathway, oxidative stress	These markers may be effective in not only the diagnosis but also the prevention and management of ischemic stroke	Insufficient sample size, limited ethnic diversity	Lee et al. (2020b)
C3, ICAM-2, PLGLA, STXBP5, IGHV3-64	Plasma	MS	Up	Single-center internal validation	90	0.98	Immune responses, complement pathway, leukocyte migration	Biomarker-based prediction models showed 75%–88% sensitivity for identifying patients with IS.	Lack of longitudinal dynamic data, insufficient sample size	Marto et al. (2023)
IGF2, LYVE1, PPBP, THBS1	Plasma	iTRAQ-LC-MS/MS	Up	Single-center external validation	60/66^*^	0.731, 0.813, 0.754, 0.769	Angiogenesis, inflammation	The 4-biomarker panel complements imaging for AIS diagnosis; IGF2, LYVE1 and THBS1 have prognostic value	Lack of longitudinal dynamic data, insufficient sample size, retrospective study	Qin et al. (2019)
GFAP, BCAN, SNAP25, SPOCK1, S100A12, MNDA	Plasma	Olink® proximity extension assays	Up	Internal validation	388	0.887, 0.820, 0.797, 0.786, 0.677, 0.657	Inflammation, apoptosis, synaptic remodeling, astroglial reactivity, ECM remodeling	Within a few hours of symptom onset, it is possible to distinguish between IS and ICH.	Lack of independent external verification and orthogonal verification techniques	Nunez-Jurado et al. (2025)

Abbreviation: C1s, complement component 1 subcomponent S; DIA, data independent acquisition; ICH, intracerebral hemorrhage; ICAM-2, intercellular adhesion molecule 2; IGHV3-64, immunoglobulin heavy variable 3–64; iTRAQ-LC-MS/MS, isobaric tags for relative and absolute quantitation-liquid chromatography-tandem mass spectrometry; MNDA, myeloid cell nuclear differentiation antigen; MRM, multiple reaction monitoring; NT-proBNP, N-terminal pro-B-type natriuretic peptide; PADI4, peptidyl arginine deiminase 4; PPBP, pro-platelet basic protein; S100A12, S100 calcium-binding protein A12; STXBP5, syntaxin binding protein 5; SWATH-MS, sequential window acquisition of all theoretical mass spectra.

* Values before and after the slash indicate “Cohort size” in the discovery cohort and validation cohort, respectively.

^†^ The “AUC” data are from the discovery cohort.

Abnormal activation of the coagulation system is a central mechanism in the pathogenesis of ischemic stroke. Proteomics analysis confirmed that, prothrombin (F2), fibrinogen alpha chain (FGA), plasminogen (PLG), histidine-rich glycoprotein (HGR), coagulation factor IX (FIX), fibronectin (FN1), vitronectin (VTN), thrombospondin 1 (THBS1), vitamin K-dependent protein S (PROS1), and many other coagulation-related proteins are significantly upregulated in ischemic stroke patients (Lee et al., 2020a; Lee et al., 2020b). The abnormal expression of these proteins directly contributes to the formation and stabilization of thrombi, suggesting that multilevel activation of the coagulation cascade is a key pathological basis for thrombus formation in ischemic stroke. In particular, the fibrinogen alpha chain is also involved in pathological processes such as oxidative stress, immune inflammation, and apoptosis (Lee et al., 2020b). Fibrinogen promotes neutrophil activation by specifically binding to the CD11b/CD18α subunits on the surface of neutrophils. These neutrophils migrate to the brain, exacerbating ischemic brain injury by releasing reactive oxygen species (ROS), proteases, and cytokines that disrupt the BBB (Lee et al., 2020b).

Post-translational modifications (PTMs) of proteins promote functional diversity by regulating protein activity, stability, subcellular localization, and protein-protein interactions; these interactions enhance gene expression and regulate cellular physiological functions. A growing body of evidence suggests that PTMs are closely associated with the pathophysiological changes in ischemic stroke. For example, adenosine triphosphate (ATP) depletion leads to a dramatic reversal in the phosphorylation status of adenosine monophosphate-activated protein kinase (AMPK) and mammalian target of rapamycin (mTOR) signaling pathways, triggering excessive activation of autophagy (Huang et al., 2020). The sharp increase in anaerobic glycolysis leads to massive local accumulation of lactate in brain tissue, triggering widespread protein lactation, which in turn affects mitochondrial cell death, mediates neuronal death, and contributes to the process of brain injury (Zhao et al., 2023). Furthermore, the regulation of PTM-mediated immune cell activation and infiltration influences brain injury following stroke. Small ubiquitin-like modifier (SUMO)ylation of annexin A1 (ANXA1) in microglia mediates the neuroprotective effects of the tat-fused nuclear translocation signal peptide (Tat-NTS) peptide against ischemia-reperfusion injury (Bian et al., 2025).

Advances in proteomics hold promise for the diagnosis and treatment of ischemic stroke. However, recent preclinical studies have tended to use transient models and focus on changes in brain-derived proteins during the acute phase of ischemic stroke. Consequently, research on permanent models and longitudinal proteomic changes remains scarce. In clinical studies, individual differences in patient response to treatment, as well as genetic and proteomic characteristics, have been overlooked. Future proteomics research, whether in clinical or preclinical settings, should focus on dynamic changes in the proteome, such as the pre-stroke phase, as well as pathological changes during the acute, subacute, and chronic phases.

#### 3.2.4 Metabolomics

Metabolomics is a discipline that involves the comprehensive qualitative and quantitative analysis of all small-molecule metabolites (typically with a molecular weight <1500 Da) within living organisms (Yong et al., 2020). Metabolic changes following ischemic stroke are primarily driven by three pathophysiological mechanisms: excitotoxicity with activation of glutamate, oxidative stress with production of free radicals, and stroke-induced inflammation affecting lipid metabolism (Sidorov et al., 2025). All of these mechanisms lead to the accumulation of metabolites in ischemic brain tissue, while disruption of the BBB allows these metabolites to diffuse into the bloodstream, thereby exacerbating brain injury. After an extensive literature review, we found only a few longitudinal studies on serum metabolomics. A recent study combining cross-sectional and longitudinal data identified the main patterns of serum metabolomic changes following acute ischemic stroke: glycerol (Gly), phosphatidylethanolamine (PE), ceramides (Cer), phenylalanine (Phe), and their derivatives are increasing, while histidine (His), tyrosine (Tyr), valine (Val), glutamine (Gln), phosphatidylcholine (PC), sphingomyelin (SM), and fatty acids (FA) are decreasing (Sidorov et al., 2025). Wang et al. observed similar trends in phenylalanine and sphingomyelin in rat model (Wang et al., 2014). Another study compared sphingosine-1-phosphate (S1P) and Cer levels between the first 48 h and the 48–72-h period following acute ischemic stroke, revealing that S1P and very long-chain ceramides were significantly reduced in acute ischemic stroke patients, whereas long-chain ceramides exhibited the opposite trend (Lee T. H. et al., 2022). However, there are still many obstacles to overcome in the clinical application of metabolomics. Most studies have not strictly matched baseline comorbidity characteristics between patient and control groups, nor have they controlled for the confounding effects of statins; limited sample types, small sample sizes, high patient heterogeneity, and inconsistent blood collection times have led to poor reproducibility of results. Table 4 provides the main potential ischemic stroke biomarkers found in metabolomics researches.

**TABLE 4:** Potential ischemic stroke biomarkers identified from metabolomics studies.

Metabolite	Sample	Cohort size	AUC	Validation status	Findings	Limitations	Refs
Ornithine, asparagine, Val citrulline, cysteine	Serum	216	0.968	No validation	These five amino acids demonstrated extremely high accuracy in the early diagnosis of mild-to-moderate ischemic stroke	Lack of validation queue, insufficient sample size	Tao et al. (2023)
Glycoursodeoxycholic acid, docosapentaenoic acid (22n-6), fatty acid hydroxy fatty acid 38:4	Serum	177/143*	0.944/0.884*	Longitudinal validation	Used to distinguish between large-artery atherosclerosis and small -vessel occlusion	Lack of longitudinal dynamic data, insufficient sample size, demographic imbalance, incomplete severity assessment	Yu et al. (2025)
Lysine, serine, threonine, kynurenine, putrescine, LPC (16:0)	Serum	193/123^†^	0.895, 0.811^†^	Internal validation	Used to distinguish between large-artery atherosclerosis and cardioembolic stroke	Lack of longitudinal dynamic data, insufficient sample size, demographic imbalance, incomplete correction of prior treatment	Lee et al. (2024)
Biliverdin, N-Acetylmuramic acid	Plasma	42	0.964	No validation	As molecular clock metabolites, the two markers can accurately differentiate acute ischemic stroke patients within 0–4.5 h and 4.5–24 h after onset, guiding thrombolytic therapy	Very small sample size, sample imbalance between groups, lack of longitudinal dynamic data	Li et al. (2025)
Acetyllysine, methionine sulfoxide, succinate/lactate/trehalose, glucuronic acid	Plasma	908/889	0.700	Prospective cohort validation	These metabolites or the composite SMS are associated with ischemic stroke and outperform traditional risk factors in risk prediction	Lack of longitudinal dynamic data, limited ethnic diversity	Balasubramanian et al. (2022)
Phenylacetylglutamine	Plasma	200/751*	0.700/0.632*	Longitudinal validation	High phenylacetylglutamine levels are independently associated with IS and an increased risk of poor short-term outcomes	Lack of multicenter validation and dietary and gut microbiome data, preclusion of causal inference by observational design, limited ethnic diversity	Yu et al. (2021)
Cer	Plasma	117	​	No validation	higher concentrations of Cer(d18:1/18:0), Cer(d18:1/20:0), and Cer(d18:1/22:0) are associated with the 3-month functional outcomes in AIS.	Lack of validation queue, inability to establish causal relationships, insufficient sample size	Lee et al. (2022b)
Gly, PE, Cer, Phe, His, Tyr, Val, Gln, PC, SM, FA	Serum	181	​	Corroborated validation	Four main patterns of metabolome changes following AIS were identified	Limited interpretation due to metabolites with unidentified chemical formula, insufficient sample size, lack of separate external queue	Sidorov et al. (2025)

Abbreviation: SMS, stroke metabolite score.

* Values before and after the slash indicate “Cohort size” or “AUC” in the discovery cohort and validation cohort, respectively.

^†^ Values before and after the slash indicate “Cohort size” or “AUC” in the training and test sets, respectively.

## 4 Integromics and systems biology

### 4.1 Multi-omics data integration strategies

With the rapid development of high-throughput technologies, including genomics, transcriptomics, epigenomics, proteomics, and metabolomics, both the volume and complexity of multi-omics data have increased substantially. Single-omics analyses are often insufficient to fully explain disease onset and progression, whereas multi-omics integration provides a more systematic biological perspective across multiple molecular layers and may facilitate disease prediction, patient stratification, and the discovery of potential biomarkers. We divide integration strategies into four major categories: statistics-based methods, network-based methods, multivariate methods, and machine learning and artificial intelligence.

#### 4.1.1 Statistical-based methods

Protein quantitative trait locus (pQTL) and eQTL analyses are representative association-based statistical approaches that link genetic variation to downstream transcriptomic and proteomic variation. MR has emerged as a particularly powerful framework for causal inference in the multi-omics toolkit for ischemic stroke research. By using genetic variants as instrumental variables, MR can assess potential causal relationships between molecular exposures, such as circulating proteins, metabolites, or gene expression levels, and stroke risk while minimizing confounding (Dichgans et al., 2019). Summary-data-based MR (SMR) further enables the integration of GWAS summary statistics with eQTL/pQTL data to identify genes whose genetically predicted expression or protein abundance is associated with stroke risk. In a multi-omics integration study, Wang et al. integrated three large-scale GWAS resources (GWAS Catalog, MEGASTROKE, and Open GWAS) with cis-eQTL and cis-pQTL data from 13 brain regions and identified 124 novel loci associated with ischemic stroke. Subsequent MR and colocalization analyses prioritized seven candidate causal genes, including CPNE1, HSD17B12, and SFXN4 (Wang M. et al., 2025).

#### 4.1.2 Network-based methods

Network-based integration methods offer a complementary framework for capturing complex regulatory relationships among molecular entities in ischemic stroke. The protein–protein interaction networks (PPIN) and ceRNA networks have been widely used to delineate regulatory landscapes in ischemic stroke. PPIN systematically depict the physical binding patterns of proteins under various physiological and pathological conditions. Within the framework of systems biology, PPIN is studied through graphical representations, in which proteins are treated as nodes and the physical (functional) associations between them are treated as edges. This approach identifies nodes with a high degree of interaction (DoI), commonly referred to as hubs. These hubs serve as major channels, and their functional integrity is central to cellular function. Therefore, computational analysis of primary neighbor and secondary interactions not only uncovers new biological connections and predicts the functions of unknown proteins but also reveals molecular modules undergoing abnormal regulation during disease pathogenesis (Basu et al., 2021). One study identified 16 proteins between ischemic stroke patients and healthy controls, 13 of which exhibited highly interconnected interaction networks. Apolipoprotein B (APOB) and serpin family a member 1 (SERPINA1), as the hub proteins with the highest Dol values in this network, may play central regulatory roles in the pathophysiological processes of ischemic stroke (Misra et al., 2022). Kong et al. recently integrated whole-blood transcriptomic data (mRNA, miRNA, and lncRNA) with DrugBank drug-target information and the STRING PPI network to construct a multilayer drug-protein-ceRNA regulatory network for acute ischemic stroke. This study identified FN1 and MMP9 as key predictive targets, which were further supported by molecular docking simulations (Kong et al., 2026). As one of the key mechanisms by which ceRNA networks exert their functions as ncRNAs, this topic has already been discussed in detail in Section 3.2.2 on transcriptomics and will not be repeated here.

#### 4.1.3 Multivariate methods

Multivariate methods represent the broadest and most diverse category among multi-omics integration strategies. They mainly extract latent variables through algebraic decomposition, capture shared patterns among omics and reduce data dimensionality, thereby identifying key associations and simplifying the integration analysis process. They include two types of tools: algorithms specifically designed for omics integration, such as sum of scores principal component analysis (SUM-PCA), method for the functional integration of spatial and temporal omics data (MEFISTO), data integration analysis for biomarker discovery using latent components (DIABLO), multi-omics factor analysis (MOFA) and its extensions, and algorithms adapted for this scope, such as principal component analysis (PCA), partial least squares (PLS), co-inertia analysis (CIA), multiple co-inertia analysis (MCIA), and MFA (Morabito et al., 2025).

#### 4.1.4 Machine learning and artificial intelligence

Machine learning has become indispensable for analyzing the high-dimensional and heterogeneous data generated by multi-omics studies of ischemic stroke. It enables computational systems to analyze complex datasets, recognize patterns, and perform prediction, classification, stratification, or decision support in an automated manner. These algorithms are generally divided into supervised and unsupervised learning, depending on whether the data include labeled outcomes, namely, the known class or outcome for each sample (Morabito et al., 2025). In supervised learning, a model learns the mapping between input features and output labels during training and applies this learned relationship to predict labels for previously unseen data. Common supervised machine learning techniques include linear regression, logistic regression, decision trees, random forests, support vector machines, and neural networks. By contrast, unsupervised learning aims to infer latent structures or patterns from input datasets that lack labeled response variables. Common unsupervised approaches include clustering methods, neighbor-based algorithms, dimensionality reduction, and anomaly detection techniques. A systematic review evaluated ML models that used multi-omics data for stroke risk stratification and reported area under the receiver operating characteristic curve (AUCs) of 0.75–0.96 for acute diagnostic models and 0.75–0.97 for models predicting stroke onset risk; the highest externally validated performance was achieved by a support vector machine trained on a metabolomics-proteomics dyad for the classification of mixed ischemic and hemorrhagic stroke (Yoo et al., 2026).

Overall, multi-omics classification models outperform single-omics models in terms of accuracy and AUC (Morabito et al., 2025). This finding underscores the value of investigating multilayer biological processes to improve our understanding of diseases and phenotypes. Nevertheless, multi-omics data are often characterized by relatively small sample sizes, high feature dimensionality, substantial heterogeneity across omics platforms, extensive missingness, batch effects, and class imbalance, all of which may increase the risk of model overfitting and poor reproducibility (Spooner et al., 2025). Therefore, the development of multi-omics classification models should incorporate cross-validation, independent validation cohorts, feature selection, data normalization, missing-value imputation or handling, and model interpretability analyses to enhance both the reliability and biological interpretability of predictive results (Morabito et al., 2025; Spooner et al., 2025).

### 4.2 Systems biology

Systems biology, as a cross-scale, multidimensional approach to integrating complex multi-omics data, has garnered widespread attention in recent years. It not only integrates molecular entities at a single scale but also focuses on interaction networks across multiple molecular levels and between different biological scales, such as cells and organs. This approach enables the analysis of complex disease phenotypes at the systems level, thereby identifying biomarkers and targets with potential for diagnosis, prognosis, and treatment (Li et al., 2022).

The pathophysiological mechanisms of ischemic stroke are extremely complex, and targeting a single pathway often fails to effectively reverse disease progression. Therefore, the use of integrated multi-omics approaches to identify relevant pathological processes in ischemic stroke and modulate them through various combination therapies is crucial for the treatment of ischemic stroke. For example, an integrated multi-omics approach has revealed the comprehensive molecular network through which lipoprotein(a) [Lp(a)] and its encoding gene, *LPA*, drive atherosclerosis in large arteries. This association, spanning from genetic variants to metabolic alterations, provides unprecedented insight into the pathogenic mechanisms of Lp(a).

GWAS studies have identified *LPA* as a significant risk locus for large artery atherosclerosis (Mishra et al., 2022). The apolipoprotein (a) [Apo(a)] encoded by *LPA* forms Lp(a) particles through covalent binding via disulfide bonds with ApoB100, which is encoded by the APOB gene (Kronenberg et al., 2022). Proteomics studies have identified APOB as a potential biomarker for ischemic stroke (Misra et al., 2022). Lp(a) is a low-density lipoprotein (LDL)-like particle whose actions adversely affect vascular inflammation, atherosclerotic lesions, endothelial function, and thrombogenesis, thereby contributing to atherosclerotic events (Lampsas et al., 2023). Furthermore, Lp(a) serves as the primary carrier of oxidized phospholipids in plasma, and certain atherogenic effects of Lp(a) are mediated by oxidized phospholipids, including chemotaxis, foam cell formation, inflammation enhancement, and plaque instability (Lampsas et al., 2023). Furthermore, oxidized phospholipids participate in the regulation of metabolic intermediates such as phosphatidylserine and triglycerides, which can be further degraded into bioactive molecules like choline and carnitine, thereby influencing neurotransmitter release and metabolic homeostasis (Tang et al., 2025). The above evidence indicates that Lp(a) plays a systemic role in the pathological progression of ischemic stroke through multi-level interactions involving the genomics, proteomics, and metabolomics. Therefore, the integration of multi-omics approaches can reveal molecular regulatory networks that link various biological processes, opening up new avenues for research into complex biological systems.

## 5 Clinical translation

The high heterogeneity of ischemic stroke poses significant challenges for its clinical management. With the rapid advancement of genetics and omics technologies, the management of ischemic stroke across its entire lifecycle is gradually shifting from a traditional “one-size-fits-all” approach toward precision medicine. This chapter systematically outlines the latest advances in the clinical translation of genetics and omics technologies across four dimensions: primary prevention, acute-phase management, secondary prevention, and prognosis of ischemic stroke.

### 5.1 Primary prevention: risk stratification and high-risk screening

Accurately identifying asymptomatic high-risk individuals is central to primary prevention of ischemic stroke. As GWAS continue to advance, PRS, as a tool for quantifying individual genetic susceptibility, have demonstrated significant potential in predicting the risk of ischemic stroke. Recently, researchers have been continuously refining methods for constructing PRS and integrating them with clinical and multi-omics to achieve more precise risk stratification.

Early clinical translation of single PRS models faced two major bottlenecks: insufficient predictive performance and poor cross-population transferability (Kachuri et al., 2024). A study generated a composite PRS (metaPRS) integrating clinical risk factors and cardiovascular profiles for Chinese individuals, whose association with incident stroke was further externally verified in the large prospective China-PAR cohort with a maximum 9-year follow-up (Lu et al., 2021). Individuals with high genetic risk had approximately twice the risk of events compared to those with low genetic risk, and good cardiovascular health significantly mitigated the absolute risk burden associated with high genetic risk. It significantly improved prediction accuracy. GIGASTROKE study developed an integrated polygenic risk score (iPGS) model that combines trans-ancestral and specific features, based on ultra-large-scale multi-ancestry GWAS data (Mishra et al., 2022). This model demonstrated strong predictive power for ischemic stroke across European, East Asian, and African populations. Its predictive performance is independent of traditional clinical risk factors and effectively addresses the poor across ancestry transferability of previous PRS models caused by training data being limited to European populations.

Integrating PRS with clinical risk factors improves the prediction of ischemic stroke risk. Recent evidence suggests that adding the Combined PGS for ischemic stroke and its associated conditions—such as AF, venous thromboembolism, coronary artery disease, hypertension, and Type 2 diabetes—to existing clinical risk factor models significantly enhances stroke risk assessment and facilitates better screening of high-risk individuals (Huang et al., 2024). A study proposed an innovative “multiplicative model” (SCORE2 × PRS-factor) by combining PRS with guideline-recommended cardiovascular disease risk prediction tools, and completed external validation using independent population cohorts (Li et al., 2024). It confirmed that the relative genetic risk reflected by PRS (PRS-factor) remains highly stable across different clinical absolute risk strata and exhibits independent effects from clinical risk factors. By multiplying SCORE2 by the PRS-factor, this model successfully reclassified 9.55% of “moderate-risk” individuals in the UK Biobank cohort as “high-risk,” increasing the total number of high-risk individuals by 56.6%. More importantly, the actual incidence of cardiovascular events among these individuals “upgraded” to high risk by PRS was twice that of the unreclassified intermediate risk individuals and was very close to the incidence rate of the original high-risk group (9.13%). Similarly, the PRS factor reclassified 8.29% of participants from intermediate to high risk within the Framingham and ARIC cohorts (Li et al., 2024). This evidence strongly supports the latest clinical consensus of the European Society of Cardiology: incorporating PRS as a multiplier factor into existing clinical risk assessment frameworks can accurately identify individuals who are clinically intermediate-risk but genetically high-risk, thereby guiding the early initiation of more aggressive primary prevention interventions (Schunkert et al., 2025).

Merging polygenic risk scores (PRSs) with multi-omics data can significantly improve the predictive accuracy for ischemic stroke, as exemplified by summed transcriptome-polygenic risk scores (STPRSs) (Cai et al., 2025) and ischemic stroke protein risk score (IS-ProRS) (Gan et al., 2025). Their predictive performance was significantly superior to that of traditional clinical models and single-gene PRS models. They facilitate a better appreciation of the genetic and molecular determinants of stroke and offers the promise of more individualized preventive and treatment strategies.

Notably, most of the existing evidence still comes from retrospective cohorts, cross-sectional data or foundational case-control models, rather than practical clinical trials capable of demonstrating that feedback of PRS results can alter physician behavior, patient adherence, treatment intensity, and stroke outcomes. Furthermore, PRS studies remain inconsistent in terms of reporting transparency, with variations in the study population, selection of genetic loci, calibration methods, ancestral correction, and validation metrics. This makes it difficult to compare different models and to define clinically actionable risk thresholds. Therefore, before PRS is used for stroke screening or recurrence prediction, stroke care systems should require external validation across different healthcare settings and prospective longitudinal evaluation of clinical utility, while standardizing the reporting of metrics such as model performance, reclassification ability, calibration, and receiver operating characteristic (ROC) curve analysis (Abraham et al., 2021). Furthermore, since ischemic stroke is a complex disease resulting from the combined effects of genetic susceptibility, lifestyle, clinical risk factors, the social environment, and external exposures, gene-environment interactions should also be systematically evaluated.

### 5.2 Acute-phase management: precise diagnosis and personalized treatment

#### 5.2.1 Identification of etiological subtypes

In the acute management of ischemic stroke, identifying the etiological subtype is crucial for developing individualized prevention strategies. Recent genetic studies have revealed the distinct genetic architectures of different stroke subtypes. Large artery atherosclerotic, cardioembolic stroke and small vessel occlusion have been shown to have partially overlapping but distinct genetic risk profiles (Yoshimoto et al., 2025). GIGASTROKE identified 11 novel loci associated with large artery atherosclerotic, including genes involved in inflammation and vascular smooth muscle cell function. 8 novel loci associated with cardioembolic stroke, including genes involved in cardiac development and function. 3 novel loci associated with small-vessel occlusion, including those involved in ECM homeostasis and endothelial function (Mishra et al., 2022).

Multi-omics have also provided powerful tools for identifying stroke subtypes. At the transcriptomics level, In the differential diagnosis of stroke subtypes, the relative expression levels of mRNA PFKFB3, lncRNA *XIST*, and microRNA-340-5p (miR-340-5p) serve as useful biomarkers for distinguishing ischemic from hemorrhagic stroke, with AUCs of 0.980, 0.990, and 0.979 (Elhorany et al., 2024). RNA-seq analysis found that large artery atherosclerosis clots were significantly enriched in genes associated with greater oxidoreduction and T-cell processes. Transcriptomes of cardioembolic clots had greater enrichment of high levels of inflammation, and clots of other determined origin were enriched for aberrant platelet and hemoglobin related processes. It indicated that transcriptomic characteristics of clots could distinguish between these subtypes (Tutino et al., 2023). Furthermore, at the proteomics level, patients with cardioembolic stroke also tend to exhibit a higher inflammatory burden than those with large artery atherosclerosis and small-vessel occlusion (Pacinella et al., 2025). A metabolomics analysis confirmed that a triple metabolite panel consisting of ursodeoxycholic acid, docosahexaenoic acid, and hydroxy fatty acids can effectively distinguish between the large artery atherosclerosis and small-vessel occlusion subtypes. ROC curve analysis confirmed that the discriminatory performance of this combination was significantly superior to that of the three metabolites used individually, with AUC values of 0.944 and 0.884 in the discovery and validation cohorts, respectively (Yu et al., 2025). Lysine, serine, threonine, kynurenine, putrescine and lysophosphatidylcholine acyl C16:0 have been identified as biomarkers capable of distinguishing between large-artery atherosclerosis and cardioembolic stroke (Lee et al., 2024). Understanding the genetic and multi-omics basis of these stroke subtypes may provide a foundation for developing precise diagnosis and personalized treatment approaches.

Etiological diagnosis based on the trial of org 10,172 in acute stroke treatment (TOAST) classification not only determines treatment strategies during the acute phase but also serves as a key clinical factor in assessing the risk of hemorrhage following endovascular treatment and intravenous thrombolysis. Studies have shown that cardioembolic stroke and atherosclerosis are independent risk factors for hemorrhage occurring within 24 h after endovascular treatment (Tong et al., 2022). Additionally, cardioembolic stroke and AF are independent predictors of all European cooperative acute stroke study hemorrhage subtypes following thrombolysis (Zeinhom et al., 2024).

#### 5.2.2 Hemorrhagic transformation

At the genetic level, polymorphisms in genes associated with hemorrhagic transformation and ECM remodeling have been shown to be closely linked to the risk of hemorrhagic transformation. Following recombinant tissue plasminogen activator (r-tPA) treatment, four shared sites (e.g., *PLXND1*, *CHD9*, *ZNF366*, *ROBO1*) were identified between hemostatic factors and the hemorrhagic transformation. This suggests that there is a common regulatory mechanism linking fibrinogen and von Willebrand factor levels to hemorrhagic transformation (Gallego-Fabrega et al., 2024). Furthermore, *MMP9* is a key pathological molecule involved in the BBB disruption, its genetic polymorphisms and elevated plasma expression levels are significantly positively correlated with the risk of tPA-associated hemorrhagic transformation (Guo P. et al., 2025).

At the multi-omics level, Transcriptomic sequencing revealed significant differences in gene expression in patients with hemorrhagic transformation regarding ubiquitin-mediated proteolysis, the MAPK signaling pathway, and the HIF-1 signaling pathway. Dysregulation of these pathways is directly involved in vascular endothelial damage and inflammatory responses (Han et al., 2022). Proteomic analysis indicates that low levels of von Willebrand factor are an independent risk factor for symptomatic hemorrhagic transformation. Incorporating this protein biomarker into clinical prediction models can significantly improve the accuracy of symptomatic hemorrhagic transformation prediction (Yuan et al., 2025). Furthermore, histidine-rich glycoprotein levels rise significantly following tPA treatment and mitigate tPA-associated hemorrhagic transformation by modulating neutrophils (Jiang et al., 2024). Targeted therapies can be tailored to these targets. For example, adding dipyridamole to aspirin therapy can reduce von Willebrand factor expression in endothelial cells (Yuan et al., 2025). Drugs such as statins, edaravone, minocycline, rosiglitazone, and clofibrate can be used to inhibit the expression or activity of MMP-9 (Guo P. et al., 2025). The use of these therapeutic agents may effectively reduce hemorrhagic transformation.

### 5.3 Secondary prevention: recurrence prediction

Accurately assessing the risk of recurrent ischemic stroke is a key prerequisite for guiding secondary prevention management and achieving personalized treatment.

At the genetic level, Recent large-scale prospective studies developed the iPRS using the China Kadoorie Biobank and the UK Biobank. The iPRS is not only a predictor of first-time onset but also an independent risk factor driving stroke recurrence and secondary coronary heart disease (Han et al., 2025). Furthermore, a metaGRS study based on BioBank Japan and specifically optimized for East Asian populations, found that the risk of stroke recurrence was significantly increased in the high genetic risk score group, and this genetically driven risk of recurrence was particularly pronounced in patients without a history of hypertension (Kojima et al., 2025). This suggests that among patient groups appearing to be “low-risk” based on traditional clinical assessments, genetic screening can accurately identify individuals with masked high recurrence risk, thereby providing a basis for initiating intensive secondary prevention at an early stage.

Integrating dynamic omics features with static clinical data is emerging as a cutting-edge strategy for improving the accuracy of recurrence prediction. The Multi-omics Prediction Model for Stroke Recurrence (MPSR) innovatively incorporates Lnet Transformer layers and a dynamic weighting mechanism to integrate clinical characteristics, imaging data, and multi-omics biomarkers from patients with ischemic stroke. Results show that this model achieved high accuracy, AUC, specificity, and sensitivity of 0.96, 0.97, 1.0, and 0.94, respectively, in predicting stroke recurrence within 1 year. Its predictive performance comprehensively surpassed that of traditional single-modality prediction tools and conventional machine learning algorithms (such as random forests and support vector machines) (Miao et al., 2024).

Predicting the risk of stroke recurrence can provide a basis for personalized treatment in secondary prevention; however, significant heterogeneity in individual drug responses further limits the efficacy of conventional treatments. Against this backdrop, pharmacogenomics offers a new direction for optimizing precision medication in secondary prevention by elucidating the regulatory role of genetic polymorphisms in drug metabolism and pharmacodynamics.

The CHANCE-2 trial demonstrated that clopidogrel fails to activate effectively in patients with mild ischemic stroke or transient ischemic attack who carry a loss-of-function *CYP2C19* allele, resulting in extremely high residual platelet reactivity. Replacing clopidogrel with the non-prodrug ticagrelor (in combination with aspirin) significantly reduces the risk of stroke recurrence within 90 days (Wang et al., 2021). Based on this robust evidence, the CPIC guidelines have formally recommended incorporating *CYP2C19* genotyping into clinical decision-making pathways for clopidogrel. However, the guidelines also note that clinical variables such as cost-effectiveness, adherence, polypharmacy, age, diabetes, obesity, and smoking are not within the core scope of the guidelines, suggesting that clinical implementation still requires localized processes and multi-factor decision support (Lee C. R. et al., 2022). Although there is strong evidence supporting several gene-drug associations, their clinical adoption has been slow. The main reasons include inconsistencies in research findings across different settings, a lack of stroke-specific randomized trials, and practical and cost-effectiveness issues related to implementing genetic testing in clinical practice (Lee et al., 2026). Detailed research progress on the clinical application of pharmacogenomics in stroke management has already been summarized in comprehensive reviews; this paper will not elaborate further on this topic (Lee et al., 2026).

### 5.4 Post-stroke rehabilitation and long-term prognosis assessment

GWAS have revealed that specific genetic polymorphisms have a significant impact on the prognosis of ischemic stroke. A recent GWAS studies of three distinct recovery phenotypes in mild ischemic stroke with 2-year follow-up identified novel suggestive gene-impairment associations for cognitive function (*CAMK2D*, *EVX2*, *LINC0143*, *PTPRM*, *SGMS1*, and *SMAD2*), motor (*ACBD6*, *KDM4B*, *MARK4*, *PTPRS*, *ROBO1*, and *ROBO2*), and overall impairment (*MSR1* and *ROBO2*) (Aldridge et al., 2024). Regarding prognosis, a whole-transcriptome analysis of the CLEAR trial cohort identified 467, 526, and 571 genes associated with an adverse 90-day mRS outcome at 3 h, 5 h, and 24 h after onset, respectively; Adverse outcomes were associated with upregulation of inflammatory and innate immune pathways, including IL-6, IL-1, STAT3, S100A12, MMP9, NF-κB, and Toll-like receptors (Amini et al., 2023). The prognostic value of the three proteins insulin-like growth factor 2 (IGF2), lymphatic vessel endothelial hyaluronic acid receptor 1 (LYVE1), and THBS1 has been demonstrated in predicting functional outcomes associated with improved collateral circulation (Qin et al., 2019).

In the field of post-stroke complications, genomic studies have demonstrated that the *BDNF* Val, *BDNF* Val66Met, *CST3*, and *APOE* ε4 were described to be significantly associated with post-stroke cognitive impairment (Lu et al., 2024). Transcriptomic studies indicate that specific non-coding RNAs (such as miR-21, miR-132, and miR-195) play a key role in regulating angiogenesis and synaptic plasticity, and their expression imbalances can serve as early diagnostic signals for post-stroke cognitive impairment (Lu et al., 2024). Proteomics has identified core effector proteins associated with the coagulation cascade (e.g., serum amyloid A, GP1BA) and oxidative stress (e.g., myoglobin, hemoglobin) (Lu et al., 2024), further confirming the central role of microvascular thrombosis and oxidative damage in post-stroke cognitive impairment. In terms of metabolomics, higher concentrations of Cer(d18:1/18:0), Cer(d18:1/20:0), and Cer(d18:1/22:0) are associated with the 3-month functional outcomes in acute ischemic stroke (AUC = 0.632) (Lee T. H. et al., 2022). Very-long-chain ceramides have a protective effect; the decrease in the levels of the very-long-chain ceramides Cer(d18:1/22:0) and Cer(d18:1/24:0) may be caused by changes in the activity of ceramide synthase 2, which is responsible for their synthesis; Downregulation of this enzyme’s expression can induce autophagy, which is also a key mechanism promoting stroke recovery (Lee T. H. et al., 2022). These molecular biomarkers provide theoretical basis and research directions for the development of targeted rehabilitation interventions. For example, anti-inflammatory therapy, neurotrophic factor supplementation, drug therapies that regulate the balance of miRNAs in the body, and dietary interventions (such as supplementation with L-carnitine, choline, etc.). However, omics research on post-stroke cognitive impairment is still in its early stages, and there are currently no approved diagnostic or predictive biomarkers. Translating preliminary research into clinical practice requires more rigorous validation, encompassing the application of specialized knowledge, the development of predictive models, and compliance with ethical and regulatory requirements.

## 6 Conclusion and prospects

The global burden of ischemic stroke remains high. Given its complex pathogenesis, which is influenced by genetic variations, environmental factors, and the regulation of multimolecular networks, precision diagnosis and treatment, as well as improved prognosis, remain major clinical challenges. This article systematically reviews the latest research advances in genetics and multi-omics in the diagnosis and treatment of ischemic stroke. It not only summarizes key findings ranging from mechanisms to clinical applications but also further clarifies the value of integrating multi-omics in advancing ischemic stroke research. Currently, there are still certain limitations in translating these studies into clinical practice: (1) Severe sample bias exists in existing genomic and omics databases; data are predominantly derived from European populations, resulting in a severe lack of representativeness of ancestral diversity. This limits the applicability of models such as PRS across ancestral populations and obscures population-specific pathological mechanisms. Future research requires the establishment of large-scale cohorts spanning different geographic regions and ethnic groups. (2) Cost-effectiveness and resource accessibility remain key factors limiting the integration of multi-omics technologies into routine stroke diagnosis and treatment workflows. When scaling up these technologies in primary care settings and resource-limited areas, practical challenges such as insufficient equipment, personnel, funding, and health insurance coverage persist. (3) There is a lack of prospective validation evidence. Existing studies have primarily focused on retrospective cohorts, cross-sectional data, case-control analyses, or single-center model development, with a lack of external and longitudinal validation. Future research should utilize prospective cohorts and clinical trials to further demonstrate whether these technologies can change physician behavior, reduce recurrence rates, lower the risk of hemorrhage, or improve functional outcomes. (4) Regulatory approval, standardization, and workflow integration remain key bottlenecks for clinical implementation. Clinical laboratories and regulatory agencies need to establish clear standards regarding analytical validity, clinical validity, and data privacy. Additionally, routine clinical practice requires the establishment of well-defined workflows covering test ordering, target populations, turnaround time, result entry, and decision implementation. Therefore, future efforts should not only aim to improve model performance but also simultaneously establish standardized implementation pathways for stroke scenarios, clarifying key aspects such as the timing of testing, target populations, result interpretation, treatment adjustments, and follow-up on treatment efficacy.
